# Supplementary material for: Development of a computer-aided design software for the quantitative evaluation of aesthetic damage
Source: PLoS One. 2019 Dec 18;14(12):e0226322. doi: 10.1371/journal.pone.0226322 (PMC6919621; doi:10.1371/journal.pone.0226322)
Supplement: S1 Code site — 1. (ZIP) [file pone.0226322.s001.zip › [EESC_jr][Projeto]Relatorio_07_21/reports/templates_zip/gentelella-master/documentation/index.html]

Gentellela Alela! | 


Skip to main content


Toggle navigation

Gentellela Alela! - Documentation

- About documentation
- Files & Directories
- Layout & structure
- Credits

# Introduction

Welcome to **Gentelella**, a responsive, flat and full featured admin template. This template is built on Bootstrap 3 framework using modern techniques like HTML5 and CSS3 to be used for backend solutions of any size.

## Support Requests

Browser support is targeted at **IE9+, Google Chrome, Mozilla Firefox, Apple Safari, Opera and all Android and iOS mobile browsers**. For browsers not in this list, support is not available.

## Documentation Scope

This documentation is targeted to explain **structures like files and directories, template, CSS and JavaScript or how you can configure** the template. Since this template is built on top of the excellent Bootstrap 3 framework, you main source of documentation is here: http://getbootstrap.com/css/

This documentation is also written in a style that assumes you already know the basics of CSS, JavaScript and HTML, so only the usage and concept of this template's elements that's going to be covered here.

## Getting Started

To get started, just browse through the demonstration pages. Use **Chrome Developer Tools (Chrome), Firebug (Firefox)** or similar plugins to analyze the code. To get a deeper understanding of the code, open the HTML and JS files and read through the commented code.

Use the `plain_page.html` and start right away building your own pages.

  
  

## Files and Directories

  
  

## Layout and structure

- Header
- Top Menu
- Side Menu
- Content
- Footer

#### Header

Header code below

```
                                        Gentellela Alela! |
```

#### Side Menu

Menu for navigation, showing profile and primary functions such as logout, full screen and settings

```
Gentellela Alela!


Welcome,

## Anthony Mutisya
```

#### Content

Main content wrapper with the heading and page content

```
### Plain Page Page subtile


Go!


## Page title Page subtile


- - Settings 1
  - Settings 2
```

  
  

## Credits and Thanks

| Name | URL |
| --- | --- |
| jQuery | http://jquery.com/ |
| jQuery Sparkline | http://omnipotent.net/jquery.sparkline/ |
| Auto resize textarea | http://www.jacklmoore.com/autosize/ |
| Nice scroll | http://areaaperta.com/nicescroll/ |
| C3 charts | http://c3js.org/ |
| Full screen editor | http://burakson.github.io/fseditor/ |
| jQuery Tags Input | https://github.com/xoxco/jQuery-Tags-Input |
| jQuery Validation Plugin | http://parsleyjs.org/ |
| Colorpicker for Bootstrap | https://github.com/xaguilars/bootstrap-colorpicker |
| bootstrap-daterangepicker | https://github.com/dangrossman/bootstrap-daterangepicker |
| bootstrap-fileupload | http://blueimp.github.io/jQuery-File-Upload/ |
| bootstrap-multiselect | https://github.com/davidstutz/bootstrap-multiselect |
| bootstrap-wysihtml5 | http://jhollingworth.github.io/bootstrap-wysihtml5/ |
| Dropzone | http://www.dropzonejs.com/ |
| Bootstrap switch | https://github.com/nostalgiaz/bootstrap-switch |
| SwitcheryJs | http://abpetkov.github.io/switchery/ |
| DataTables | https://github.com/DataTables/DataTables |
| DataTables tools | https://github.com/DataTables/TableTools |
| easy pie chart | https://github.com/rendro/easy-pie-chart |
| FullCalendar | https://github.com/arshaw/fullcalendar |
| jQuery Knob | https://github.com/aterrien/jQuery-Knob |
| select2 | https://github.com/ivaynberg/select2/ |
| jQuery-Autocomplete | https://github.com/devbridge/jQuery-Autocomplete |
| Twitter Bootstrap Wizard | https://github.com/VinceG/twitter-bootstrap-wizard |
| E-Charts | http://echarts.baidu.com/doc/example-en.html |
| Font Awesome | http://fortawesome.github.io/Font-Awesome/ |
